# Mode Selection and Spectrum Allocation in Coexisting D2D and Cellular Networks with Cooperative Precoding †

**DOI:** 10.3390/s19245417

**Published:** 2019-12-09

**Authors:** Yu-Wei Chan, Feng-Tsun Chien, Chao-Tung Yang

**Affiliations:** 1 College of Computing and Informatics, Providence University, Taichung 43301, Taiwan; ywchan@gm.pu.edu.tw; 2 Institute of Electronics, National Chiao Tung University, Hsinchu 30010, Taiwan; ftchien@mail.nctu.edu.tw; 3 Department of Computer Science, Tunghai University, Taichung 40704, Taiwan

**Keywords:** device-to-device, underlay, mode selection, signal-to-interference-plus-noise-ratio (SINR), block diagonalization (BD), zero-forcing beamforming (ZFBF), signal-to-leakage-and-noise-ratio (SLNR), interference management

## Abstract

In this paper, we investigate the mode selection strategies for a new device-to-device (D2D) pair becoming active in a network with a number of existing D2D sensors or users coexisting with cellular users in a D2D-enabled heterogeneous network. Specifically, we propose two selection rules, the signal-to-interference-plus-noise-ratio (SINR)-based and the capacity-based, combined with two sets of different precoding schemes and discuss their impacts on the system under a variety of scenarios. While the cooperative block diagonalization (BD) among the cellular users combined with the zero-forcing (ZF) precoding among D2D users can eliminate interference observed at the new D2D receiving sensor, the maximum signal-to-leakage-and-noise-ratio (SLNR) precoding is often a preferred option due to low-complexity implementations and comparable performance. We note that the two selection rules, the SINR-based and the capacity-based, considered in this paper impact on the system differently, with interesting tradeoff from different perspectives. Finally, we provide insights by simulations into the best selection among the three modes depending on a variety of use cases in the network.

## 1. Introduction

### 1.1. Background

In the past decade, we have witnessed an enormous growth of both the amount of mobile broadband traffic and the user demand for faster data access due to the fast development communication technologies, particularly in applications of smart devices or Internet-of-Things (IoT) sensors. According to the latest Cisco visual networking index, mobile Internet data traffic will reach 3.3 ZB(Zettabyte) per year by 2021 [1]. Such massive user demand for higher data rates has been pushing researchers to seek new paradigms to revolutionize the traditional communication methods of cellular networks. Device-to-device (D2D) communication is one of such paradigms that appears to be a promising component in next-generation cellular technologies [2,3,4,5,6,7,8,9]. The basic concept of D2D communication is first proposed in [10] for data exchange between peer nodes. Resembling conventional sensor networks and aiming for better spectral efficiency, D2D communication, which enables devices to communicate directly without the interaction of access points or base stations, is envisioned as a paradigm shift from a wider range cellular network to a smaller range network in local areas [11,12,13].

Traditionally, data transmissions between two users are hierarchically accomplished through the core network where the base station (BS) serves as a critical communication and control element in establishing a link between the two users. The physical layer and media access control layer functionalities are governed by the BS. While this hierarchical structure has been successful in modern centralized cellular networks, there are still occasions that a non-hierarchical cell-free structure would be preferred for more efficient communications, e.g., communications between a group of people watching a ball game in a stadium. If all the users are requesting a streaming video replaying a critical moment of the ball game, the nearby BS will be overwhelmed by the enormous data traffic. Moreover, if two users are located to each other, the data sharing between them still has to go through the core network regardless of how close these two users are.

On the other hand, for tackling the challenges discussed in the above, two nearby users or machine-type sensors with D2D communications can establish a direct communication link because of the better channel quality between them, instead of data transmissions via the core network. Apparently, a hybrid system with coexisting D2D users and cellular users is a promising and more flexible communication network. Specifically, the D2D users coexist with the cellular users using one of the following three modes [14,15]:

- Cellular mode: The D2D users are considered as cellular users where the data transmission is made possible via the BS.
- Underlay mode: The D2D users coexist with the cellular users non-orthogonally. More specifically, the D2D users may utilize the spectrum currently being used by cellular users for data transmissions. Transmissions of data between D2D users in the underlay mode, however, potentially incurs interference to the cellular users.
- Overlay mode: The D2D users coexist with cellular users in an orthogonal manner. Specifically, D2D users employ parts of the currently unused spectrum for data transmission. There is no interference imposed to the cellular in this mode.

There have been extensive studies in the literature investigating the problem of mode selection between the three modes for a given user in the network [16,17,18,19]. However, there has been little research on how to determine a proper functional mode when a new pair of users start new sessions of communications in the network or a new pair of communicating sensors turns active. Particularly, whether the D2D mode is beneficial to the new users with multiple existing D2D pairs is still an open problem worth investigation.

### 1.2. Related Work

Liu et al. published the pioneering work [18] discussing the impacts of distances between D2D users and cellular users on the mode selection rules for a D2D pair in a cellular system with a number of relaying nodes, assuming known distances between each communication devices. The problem of joint mode selection and power control in a network with only a single D2D pair and a number of cellular users is studied by Hakola et al. in [16]. Doppler et al. [17] investigate how a single D2D pair determines the optimal mode when coexisting with the cellular network in a multi-cell environment, taking into account the effects of interference from other cells. A joint mode selection and resource allocation approach with the user’s quality-of-service (QoS) guarantee is proposed by Wen et al. in [19]. A centralized optimal mode selection based on each user’s receiving signal-to-noise ratio is devised in [20] with consideration of the system’s sum-rate whereas a decentralized mode selection and resource allocation scheme can be found in [21]. The authors in [22,23] approach the problem from the perspective of energy efficiency.

In addition to the research work related to mode selection mentioned in the above, a significant amount of research efforts have also been dedicated to investigating interference management by proper resource allocation in D2D communications. In [24], the authors address the problem of interference on the D2D user equipment caused by the cellular users in the underlay mode. A hybrid fractional frequency reuse (FFR) and almost blank subframe (ABS) scheme are proposed to handle the inter-cell interference to the D2D receivers. In [25], the authors study the problem of maximizing the overall system spectrum efficiency while satisfying the rate requirements of all cellular users, in which a two-stage resource allocation scheme (comprising a subcarrier assignment with a greedy method and a power allocation algorithm with the Lagrangian dual method) is proposed to deal with the interference in the network with multiple D2D pairs sharing uplink spectrum resources with the cellular users.

In [26], the problem of resource sharing is studied with an aim to maximize the achievable rate of the D2D link while satisfying the QoS constraints for cellular users. The authors in [27,28] show that, when operating in the underlay mode, the interference between D2D users and cellular users can be controlled under carefully allocated power such that the D2D users can have improved total throughput without imposing much harmful interference to the cellular users. As a promising application of D2D communications in the area of autonomous driving, the authors in [29] show their research work about spectrum sharing and power allocation in a vehicular ad hoc network (VANET), particularly for vehicle-to-vehicle (V2V) links.

### 1.3. Research Objective

We can see that much work has been proposed in the D2D-enabled networks regarding mode selection and/or resource allocation, but very few discussions can be found about devising a guideline to mode selection for a new pair of D2D users becoming active the coexisting heterogeneous network with a number of D2D pairs already active in proximate locations. In this paper, we consider a network with coexisting cellular users and D2D users and focus on determining an appropriate mode, from the three basic modes, for a new D2D pair.

Interference management is critical when existing D2D users underlay among the cellular users as the D2D users utilize the spectrum currently in use by the cellular users for data transmission. To handle the problem of interference in D2D networks, cooperative precoding strategies exploiting spatial diversity by weighting the information streams from different D2D users have recently been justified as enabling approaches to significantly improve the performance in practical applications [30,31,32]. Specifically, we study the effects of two sets of precoding schemes, both capable of controlling the interference resulting from the current active D2D users and cellular users. The first set of precoding schemes we study is to adopt the block diagonalization (BD) precoding cooperatively among the cellular users and the zero-forcing (ZF) precoding cooperatively among the D2D users. To alleviate the constraints to the number of antennas when using the ZF precoding, we also consider the other set of precoding schemes by employing the maximum signal-to-leakage-and-noise-ratio (SLNR) precoding for both the cellular users and D2D users. On the other hand, when operating in the overlay mode, D2D users utilize resource blocks orthogonal to those of the cellular users, under which the cellular users experience potential interference only from the other cellular users within the cell. In this case, we consider that the cellular users employ precoding schemes, such as the BD and SLNR for controlling the interference. With these interference mitigation mechanisms, we determine the appropriate mode selection rule for a new D2D pair by evaluating the SINR of the D2D receiver and the sum capacity of the whole network.

### 1.4. Contribution

The main contributions of this work are summarized as follows:

- We propose two mode selection rules, namely the SINR-based and the sum capacity-based mode selection, to determine the functional mode for a new D2D user who has just become active in a D2D network with multiple existing D2D and cellular users.
- We study the effects of two sets of cooperative precoding schemes on the mode selection strategies. In the first set of precoding schemes, we employ the BD precoding for the cellular users and ZF precoding for the D2D users which have been proven effective in controlling the interference but with limitations in the number of antennas. In the second set of precoding scheme, we employ the SLNR precoding for both the cellular users and D2D users to alleviate the constraints in the number of antennas while capable of adequately managing the interference in the network.
- While SLNR precoding has been well studied in the literature, its application on the design of mode selection strategies in D2D networks has not been discussed. In this work, in addition to the SINR-based mode selection with the BD and ZF precoding [33], we present the first investigation about sum capacity-based mode selection strategies with the SLNR precoding.

The rest of the paper is organized as follows. Section 2 first formulates the system model for a D2D network coexisting with cellular users. In Section 3, we analytically derive the BD, ZF, and SLNR precoders for interference mitigation. The mode selection problem is explicitly described and the mode selection criterion is elaborated in Section 4. Finally, simulation results are presented in Section 5 and the conclusions are made in Section 6.

## 2. System Model

In this work, we consider the case that a new D2D pair becomes active in the network which consists of one BS and *N* cellular users coexisting with *M* pairs of D2D users, where the cellular and D2D users share the uplink (UL) spectrum for data transmission.

The BS is equipped with $N_{B}$ antennas, each cellular user’s device is equipped with at least one antenna, and each D2D user is with a single antenna. Let the total number of transmit antennas of the cellular users be $M_{t}$, which is the sum of the number of antennas of the active cellular users.

All channels are assumed to be flat Rayleigh fading with path loss. Specifically, the channel coefficient from transmitter *i* to receiver *j* is expressed by $h_{j,i}=h_{j,i}^{o}\cdot d^{\frac{-v}{2}}$, where $h_{j,i}^{o}$ is complex Gaussian with CN(0,1) and *v* is the path loss exponent. We assume that channel state information (CSI) between the BS and the cellular users and between the D2D pairs is available at the BS and at the cellular users to allow for the precoding in a centralized and coordinated fashion.

### 2.1. The Underlay Mode

**Figure 1:**
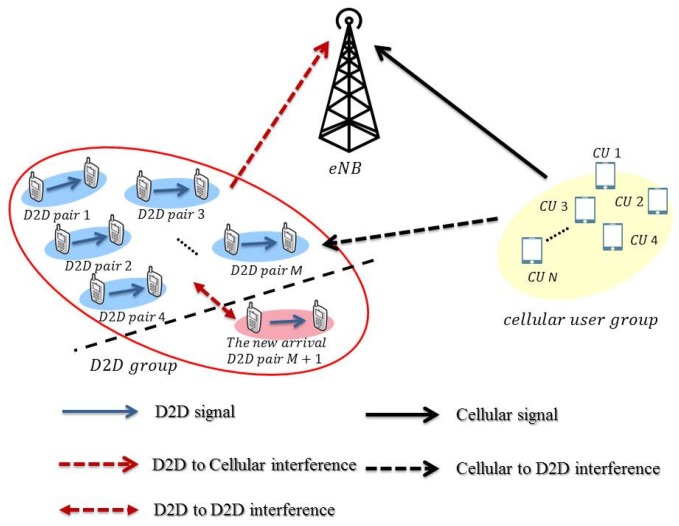
The system illustration with multiple cellular users and device-to-device (D2D) users coexisting in the underlay mode.

We illustrate a possible scenario in Figure 1 for the underlay mode. We denote the new D2D pair as the (M+1)th pair in the D2D network. When the new pair of D2D users is operating in the underlay mode, we can represent the (M+1) received signals of all the D2D receivers as

(1) $$y=H_{d}W_{d}s_{d}+H_{c}W_{c}s_{c}+n,$$

where the first term and the second term on the right-hand side (RHS) refer to the signal components from the D2D users and from the cellular users, respectively. Specifically, $y={\left[y_{1},y_{2},\ldots ,y_{M+1}\right]}^{T}$ is the vector of received signals of D2D users, the (M+1)×(M+1) matrix $H_{d}={\left[h_{1},h_{2},\ldots ,h_{M+1}\right]}^{T}$ encloses the channel coefficients between the D2D users with $h_{i,j}$ being the channel of the *j*th D2D transmitter and the *i*th D2D receiver, and $W_{d}=\left[w_{1},w_{2},\ldots ,w_{M+1}\right]$ contains the precoding vectors for all M+1 D2D users. The signal vector $s_{d}={\left[s_{1},s_{2},\ldots ,s_{M+1}\right]}^{T}$ is the transmitted data from the M+1 D2D senders with zero mean and covariance matrix $P_{d}$. The second term on the RHS is structurally identical to the first term, so $H_{c}\in C^{\left(M+1\right)\times M_{t}}$ is the channel matrix between the cellular users and the D2D receivers, $W_{c}=\left[w_{c,1},w_{c,2},\ldots ,w_{c,M+1}\right]$ refers to the matrix of precoding vectors for the cellular users, and $s_{c}={\left[s_{c,1},s_{c,2},\ldots ,s_{c,N}\right]}^{T}$ is the data of the cellular users with zero mean and covariance matrix $P_{c}$. The Gaussian noise $n={\left[n_{1},n_{2},\ldots ,n_{\left(M+1\right)}\right]}^{T}$ has zero mean and covariance matrix $\sigma^{2}I$.

### 2.2. Overlay Mode

**Figure 2:**
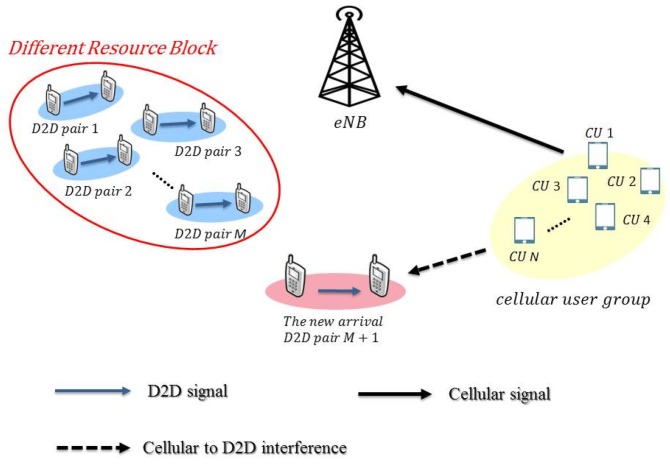
The illustration for a system with coexisting cellular users and D2D users in the overlay mode.

We illustrate a possible scenario in Figure 2 for the overlay mode. Again, the (M+1)th pair denotes the new D2D pair in the D2D network coexisting with the cellular network. When the new pair of D2D users is operating in the overlay mode, we can represent the received signals of all M+1 D2D receivers as

(2) $$y_{M+1}=h_{d_{M+1},d_{M+1}}s_{\left(M+1\right)}+h_{d_{M+1},c}^{T}W_{c}s_{c}+n_{o},$$

where $h_{d_{M+1},d_{M+1}}\in C$ is the complex Gaussian fading channel coefficient in the link of the new D2D pair, $h_{d_{M+1},c}\in C^{N\times 1}$ is the vector of complex Gaussian channel coefficients between the cellular users and the new D2D receiver, and $n_{o}$ is Gaussian noise with zero mean and variance $\sigma_{o}^{2}$. In this paper, we study the mode selection with a variety of precoding options for $W_{c}$ and $W_{d}$, and investigate mode selection rules for the new D2D pair.

## 3. Cooperative Precoding

In the underlay mode, cooperative precoding facilitates interference mitigation as the resources are shared among D2D and cellular users non-orthogonally. In this section, we discuss the BD and ZF precoders in the underlay mode.

### 3.1. BD and ZF Precoding

We first discuss the design of a BD precoder [34], which attempts to nullify the interference from the cellular users. More specifically, the BD precoding matrix $W_{c,1}\in U\left(M_{t},N\right)$ is a unitary matrix that satisfies

(3) $$H_{c}W_{c,1}=0$$

so that the second term on the RHS of (1) vanishes. From (3) we see that a basis in the null space of can be used to represent the columns of $W_{c,1}$.

Let the rank of the matrix $H_{c}$ be ${\tilde{L}}_{c}$. We can decompose $H_{c}$ using the singular value decomposition (SVD) into

(4) $$H_{c}={\tilde{U}}_{c}\left[{\tilde{\Lambda }}_{c},0_{{\tilde{L}}_{c}\times \left(M_{T}-{\tilde{L}}_{c}\right)}\right]{\left[{\tilde{V}}_{c}^{\left(1\right)},{\tilde{V}}_{c}^{\left(0\right)}\right]}^{†},$$

where the diagonal entries in ${\tilde{\Lambda }}_{c}=$ $\mathrm{diag}(\lambda_{1,c},\ldots ,\lambda_{{\tilde{L}}_{c},c})$ are the singular values of $H_{c}$ and the columns of ${\tilde{V}}_{c}^{\left(0\right)}$ constitutes a linearly independent basis of the null space of $H_{c}$. Thus, we can represent each column in $W_{c1}$ that follows the condition in (3) as a linear combination of the basis, i.e., the columns of ${\tilde{V}}_{c}^{\left(0\right)}$. Note that the necessary condition for a non-empty null space, which leads to perfect nullification of interference as shown in (3), is

(5) $$M_{T}>M+1,$$

which suggests a minimum required number of transmit antennas. To account for the number of all cellular users’ antennas, we can rewrite the condition in (5) by

(6) $$\sum_{i=1}^{N}n_{i}>M+1,$$

where $n_{i}$ denotes the number of antennas of the *i*th cellular user.

On the other hand, the interference from the existing D2D users is mitigated using the zero-forcing precoding. The ZF precoding implements linear processing on the transmitter side which completely removes the interference observed at the receiver. More specifically, the ZF precoding matrix is

(7) $$W_{d,1}=H_{d}^{H}{\left(H_{d}H_{d}^{H}\right)}^{-1}.$$

While ZF precoding is capable of eliminating the clearly specified interference, unaccounted interference may yield dramatic performance loss in the SINR. Besides, a well-known limitation of the ZF precoding is that the number of transmit antennas needs no less than the total number of antennas of all users.

### 3.2. SLNR Precoding

In this subsection, in an attempt to overcome the limitations entailed with the ZF precoders, we study a transmit precoding scheme based on maximizing the ratio of the intended signal power to the leakage power plus noise (SLNR), which does not pose any requirement on the number of transmit/receive antennas, as is suffered by the ZF precoding scheme [35], and accounts for the noise effect into the design of the precoding matrix. The leakage is defined as the interference observed by unintended users that is caused by the signal sent to the intended user, and therefore the precoding method attempted to control the signal leakage can be considered as an altruistic approach by lessening the effect on impeding others. A typical way to determine the precoding matrix is by maximizing the SLNRs of all users simultaneously [35]. Note that we use $W_{c,2}$ and $W_{d,2}$ to represent $W_{c}$ and $W_{d}$, respectively, in (1) in the following derivations for clarity.

The SLNR resulted from the *k*th D2D transmit/receive pair can be represented by

(8) $${\mathrm{SLNR}}_{k}=\frac{{\left\|h_{k}^{T}w_{k}\right\|}^{2}}{\sigma_{k}^{2}+{\left\|{\tilde{H}}_{k}w_{k}\right\|}^{2}},$$

where $\sigma_{k}^{2}$ is the noise variance observed at the receiver of the *k*th D2D pair and

(9) $${\tilde{H}}_{k}={\left[h_{1},\cdots ,h_{k-1},h_{k+1},\cdots ,h_{M+1}\right]}^{T}$$

is the shrunk channel matrix from $H_{d}$ that excludes $h_{k}^{T}$ only. It can be shown that the precoding vector for the *k*th D2D pair is given by [36]

(10) $$w_{d,2,k}^{opt}\propto \max\mathrm{ev}({\left(\sigma_{k}^{2}I_{M+1}+{\tilde{H}}_{k}^{H}{\tilde{H}}_{k}\right)}^{-1}h_{k}^{H}h_{k}),$$

where ev(A) is the eigenvector corresponding to the largest eigenvalue of the matrix A and $I_{M+1}$ denotes identity matrix with dimension (M+1)×(M+1).

**Figure 3:**
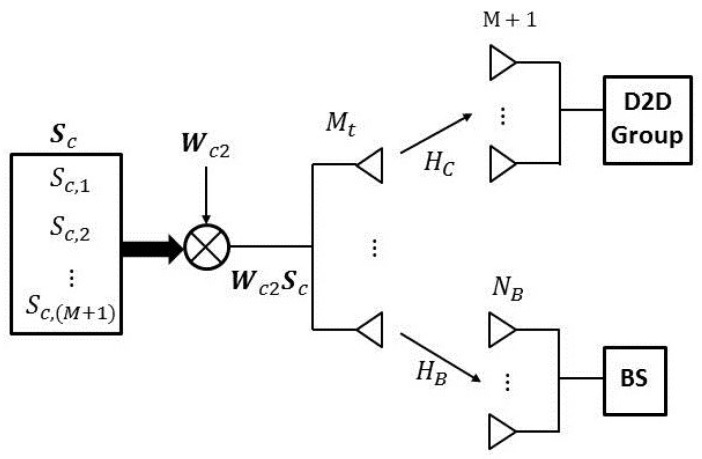
The block diagram of a multiple antenna transmission system employing beamforming with multiple streams.

We can extend the multiuser SLNR-based precoding obtained in (10) to a multi-stream precoder for the cellular users $W_{c,2}$. All the cooperating cellular users can be considered as a virtual single user but with multi-stream and the group of D2D pairs and BS are viewed as two receivers. The block diagram for multi-stream precoding is illustrated in Figure 3, where $H_{B}\in C^{N_{B}\times M_{t}}$ specifies the complex Gaussian channel coefficients between the cellular users and the BS. Then, the SLNR for cellular users is

(11) $${\mathrm{SLNR}}_{c}=\frac{E[s_{c}^{H}W_{c2}^{H}H_{B}^{H}H_{B}W_{c2}s_{c}]}{N_{B}\sigma_{c}^{2}+E\left[s_{c}^{H}W_{c2}^{H}H_{c}^{H}H_{c}W_{c2}s_{c}\right]},$$

where $\sigma_{c}^{2}$ is the noise variance of cellular users. We further evaluate the expectations in (11). It follows that

(12) $$\begin{array}{cc} {\mathrm{SLNR}}_{c} & =\frac{\mathrm{Tr}((W_{c2}^{H}H_{B}^{H}H_{B}W_{c2})}{N_{B}\sigma_{c}^{2}+\mathrm{Tr}((W_{c2}^{H}H_{c}^{H}H_{c}W_{c2})}, \end{array}$$

(13) $$\begin{array}{cc} \; & =\frac{\mathrm{Tr}((W_{c2}^{H}H_{B}^{H}H_{B}W_{c2})}{\mathrm{Tr}((W_{c2}^{H}((N_{B}\sigma_{c}^{2}I_{M_{t}}+H_{c}^{H}H_{c})W_{c2})}, \end{array}$$

where we have assumed $E[s_{d}s_{d}^{H}]=I$ and use $\mathrm{Tr}(W_{c2}^{H}W_{c2})=1$ in the derivation. The precoding matrix $W_{c,2}$ is designed based on

(14) $$W_{c2}^{opt}=\arg\max_{W_{c2}\in C^{M_{t}\times N}}{\mathrm{SLNR}}_{c}.$$

As $H_{B}^{H}H_{B}$ is Hermitian and $\left(N_{B}\sigma_{c}^{2}I_{M_{t}}+H_{c}^{H}H_{c}\right)$ is Hermitian and positive semidefinite, by generalized eigenvalue decomposition, there exists an invertible $M_{t}\times M_{t}$ matrix $T_{c}$ such that

(15) $$T_{c}^{H}H_{B}^{H}H_{B}T_{c}=\Lambda_{c}=\mathrm{diag}(\lambda_{1},\ldots ,\lambda_{M_{t}})$$

(16) $$T_{c}^{H}(N_{B}\sigma_{c}^{2}I_{M_{t}}+H_{c}^{H}H_{c})T_{c}=I_{M_{t}}$$

with $\lambda_{1}\geq \lambda_{2}\geq \ldots \geq \lambda_{M_{t}}\geq 0$. The columns of $T_{c}$ and the diagonal entries of $\Lambda_{c}$ are the generalized eigenvectors and eigenvalues of the pair $\left\{H_{B}^{H}H_{B},N_{B}\sigma_{c}^{2}I_{M_{t}}+H_{c}^{H}H_{c}\right\}$, respectively. It is then shown in [35] that the optimal precoder can be obtained by extracting the leading *N* columns of $T_{c}$ as

(17) $$W_{c2}^{opt}=\rho T_{c}[\begin{array}{c} I_{N\times N}\\ 0 \end{array}],$$

where ρ is a scaling factor so that $\mathrm{Tr}(W_{c2}^{H}W_{c2})=1$.

## 4. Mode Selection

In this section, we develop a criterion of determining a suitable mode for a new pair of D2D users based on the SINR and the system sum capacity. Particularly, the impacts of each rule on the SINR and sum capacity are investigated under the scenarios of the existing D2D pairs being in the underlay mode or the overlay mode.

### 4.1. Mode Selection by USER’s SINR

#### 4.1.1. Scenario I: Underlay Mode for the Existing D2D Users

Consider the scenario that the *existing* D2D pairs are in the underlay. The SINR of the receiver in the new D2D pair operating also in the underlay mode can be represented by

(18) $${\mathrm{SINR}}_{UN,un}=\frac{P_{new}}{E[{\left|n_{M+1}\right|}^{2}]+I_{d}+I_{c}}=\frac{{\left\|h_{M+1}^{H}w_{M+1}\right\|}^{2}}{\sigma^{2}+Mh_{M+1}^{H}W^{\prime }W^{\prime H}h_{M+1}+h_{c,M+1}^{H}w_{c,M+1}w_{c,M+1}^{H}h_{c,M+1}},$$

where $W^{\prime }=\left[w_{1},w_{2},\cdots ,w_{M}\right],\;s^{\prime }={\left[s_{1},s_{2},\cdots ,s_{M}\right]}^{T}$. The capital “*UN*" in the subscript means the current active D2D pairs are in the underlay mode whereas the small “*un*” specifies that the new D2D pair is also in the underlay mode. The terms $I_{d}$ and $I_{c}$ respectively stand for the interference from the current active D2D pairs and the cellular users.

Next, the SINR of the receiver in the new D2D pair in the overlay mode and in the cellular mode, with the existing D2D users operating in underlay, can be respectively represented by

(19) $$\begin{array}{c} {\mathrm{SINR}}_{UN,ov}\\ \;=\frac{P_{new}}{E\{{\left|n_{M+1}\right|}^{2}\}}\\ \;=\frac{E\{{\left|h_{M+1}^{H}w_{M+1}s_{M+1}\right|}^{2}\}}{\sigma_{o}^{2}}\\ \;=\frac{h_{M+1}^{H}w_{M+1}E\left\{s_{M+1}^{2}\right\}w_{M+1}^{H}h_{M+1}}{\sigma_{o}^{2}}\\ \;=\sigma_{o}^{-2}h_{M+1}^{H}w_{M+1}w_{M+1}^{H}h_{M+1}, \end{array}$$

(20) $$\begin{array}{c} {\mathrm{SINR}}_{UN,cel}\\ \;=\frac{\gamma_{ul}\gamma_{dl}}{\gamma_{ul}+\gamma_{dl}+1}\\ \;=\frac{\frac{E\{{\left\|H_{d,B}w_{M+1}s_{M+1}\right\|}^{2}\}}{E\{{\left|n_{M+1}\right|}^{2}\}}\cdot \frac{E\{{\left|h_{B,d}^{H}H_{d,B}w_{M+1}s_{M+1}\right|}^{2}\}}{E\{{\left|n_{M+1}\right|}^{2}\}}}{\frac{E\{{\left\|H_{d,B}w_{M+1}s_{M+1}\right\|}^{2}\}}{E\{{\left|n_{M+1}\right|}^{2}\}}+\frac{E\{{\left|h_{B,d}^{H}H_{d,B}w_{M+1}s_{M+1}\right|}^{2}\}}{E\{{\left|n_{M+1}\right|}^{2}\}}+1}\\ \;=\frac{H_{d,B}w_{M+1}w_{M+1}^{H}H_{dB}^{H}\cdot h_{Bd}^{H}H_{dB}w_{\left(M+1\right)}w_{M+1}^{H}H_{dB}^{H}h_{Bd}}{H_{dB}w_{M+1}w_{M+1}^{H}H_{dB}^{H}+h_{Bd}^{H}H_{dB}w_{M+1}w_{M+1}^{H}H_{dB}^{H}h_{Bd}+E\{{\left|n_{M+1}\right|}^{2}\}}, \end{array}$$

where the $\gamma_{ul}$ and $\gamma_{dl}$ are the SNR in the new D2D pair in the uplink and downlink cases, respectively, with $P_{s_{ul}}$ and $P_{s_{dl}}$ being the uplink and downlink received powers, $H_{d,B}\in C^{N_{B}\times \left(M+1\right)}$ and specify the channel effects between the BS and the transmitter in the new D2D pair and between the BS and the receiver in the new D2D pair, respectively. Having obtained the SINRs of the new D2D users in (18), (19), and (20), the mode can be determined according to

(21) $${\mathrm{Mode}}_{UN}=\arg\max_{mode}{\mathrm{SINR}}_{UN,mode},$$

where mode∈{un,ov,cl} with the abbreviations within the set clearly standing for the underlay, overlay, and cellular mode, respectively.

#### 4.1.2. Existing D2D Users in Overlay

When the current active D2D pairs are in the overlay mode, the SINRs of the receiver in the new D2D pair operating in the three modes can be represented by

$$\begin{array}{c} {\mathrm{SINR}}_{OV,un}\\ \;=\frac{P_{new}}{E\{{\left|n_{M+1}\right|}^{2}\}+I_{c}}\\ \;=\frac{E\{{\left|h_{M+1}^{H}w_{M+1}s_{M+1}\right|}^{2}\}}{E\{{\left|n_{M+1}\right|}^{2}\}+E\{{\left|h_{c,M+1}^{H}w_{c,M+1}s_{c,M+1}\right|}^{2}\}}\\ \;=\frac{h_{M+1}^{H}w_{M+1}w_{M+1}^{H}h_{M+1}}{E\{{\left|n_{M+1}\right|}^{2}\}+h_{c,M+1}^{T}w_{c,M+1}E\left\{s_{c,M+1}^{2}\right\}w_{c,M+1}^{H}h_{M+1}}\\ \;=\frac{h_{M+1}^{H}w_{M+1}w_{M+1}^{H}h_{M+1}}{\sigma^{2}+h_{c,M+1}^{H}w_{c,M+1}w_{c,M+1}^{H}h_{c,M+1}}, \end{array}$$

$${\mathrm{SINR}}_{OV,ov}={\mathrm{SINR}}_{UN,ov},$$

$${\mathrm{SINR}}_{OV,cel}={\mathrm{SINR}}_{UN,cel}.$$

Then, the mode can be determined according to

(22) $${\mathrm{Mode}}_{OV}=\underset{mode}{\mathrm{argmax}}{\mathrm{SINR}}_{OV,mode}.$$

The SINR-based selection rule focuses on the condition of the new D2D pair which indeed benefits the user. However, this rule does not account for other users in the cell. In view of the whole network, we discuss the mode selection rule from the perspective of the system sum capacity in the next subsection.

### 4.2. Mode Selection by Sum Capacity

**Figure 4:**
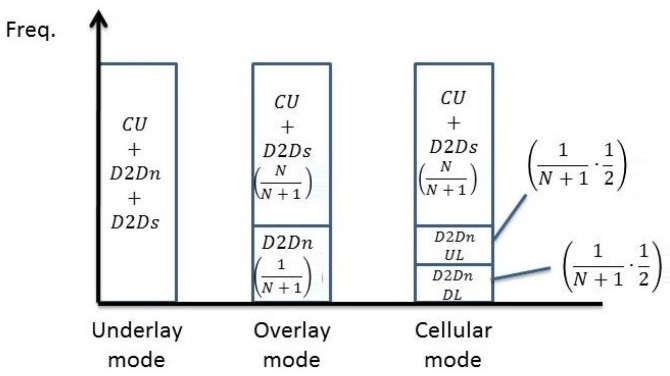
The spectrum allocation in three different modes with existing D2D pairs operating in the underlay mode.

We first consider the system where the current active D2D pairs are in the underlay mode. The new pair of D2D users can choose one of the three modes determined by the selection rule. The spectrum allocation for all users in the system is illustrated in Figure 4, where CU, D2Dn, and D2Ds respectively refer to the cellular users, the new D2D pair, and the existing D2D pairs. The allocation depends mainly on the number of new D2D users and cellular users’ antennas. When the new D2D pair coexists in the underlay fashion, all the D2D pairs share the whole spectrum non-orthogonally with the cellular users, leading to interwoven interference. On the other hand, the new D2D pair coexists in the overlay fashion, existing D2D users use the spectrum with cellular users but the new D2D pair uses the remaining non-overlapping spectrum. Finally, when the new D2D pair coexists in the cellular fashion, the spectrum allocation can be the same with the overlay mode, however, half of the spectrum allocated to the new D2D pair is for uplink and the other part is for downlink.

**Figure 5:**
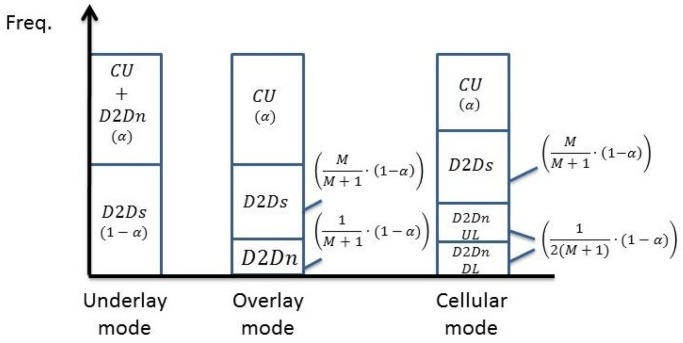
The spectrum allocation in three different modes with existing D2D pairs operating in the overlay mode.

On the other hand, for the existing D2D pairs being in the overlay mode, the spectrum allocation for all users in the cell is illustrated in Figure 5, where α denotes the proportion of spectrum used by cellular users, 0<α<1. In the simulation, we will compare the result with different values of α. When the new D2D pair coexists in the underlay fashion, it shares the spectrum with the cellular users non-orthogonally whereas the other existing D2D pairs take up the remaining part of the resources. When the new D2D pair coexists in the overlay mode, cellular users, the current active D2D pairs and a new pair of D2D users use the spectrum orthogonally so that no interference is present. When the new D2D pair is in the cellular mode, spectrum allocation is the same as the overlay mode but half of the spectrum allocated for new D2D pair is for the uplink and the other part is for the downlink.

Although the SINR-based selection rule is a fairly good choice for the new D2D users, it is not necessarily a better option for the entire system as a whole. Depending on the sum-rate of the cell, the capacity-based selection rule selects the mode with the highest sum capacity for the new D2D pair. The sum capacities of the entire network with the scenario of the new D2D pair operating in the three different modes and the current active D2D pairs operating in the underlay mode are respectively given as follows:

- The sum capacity when the new D2D paper is in the underlay mode is given by
  (23) $$R_{u}^{u}=R_{d,M+1}+R_{c,I}^{u},$$
  where the superscript and subscript of the rate *R* respectively specify the modes of the current active D2D pairs and the new pair of D2D users, with *u*, *o*, and *c* referring to the underlay mode, overlay mode and cellular mode, respectively,
  $$R_{d,M+1}=\log(\det(I_{M+1}+\beta_{M+1}H_{d}Q_{M+1}H_{d}^{H}))$$
  is the capacity of all the D2D users with $\beta_{M+1}$ being the ratio between the transmit power and noise power and $Q_{M+1}$ being the D2D pairs’ signal covariance matrix, and
  $$R_{c,I}^{u}=\log(\det(I_{N_{B}}+\rho H_{B}Q_{c}H_{B}^{H}))$$
  is the capacity of the BS with ρ being the SINR at the BS with $Q_{c}$ being the cellular users’ signal covariance matrix. From Figure 4, the coefficient of $R_{d,M+1}$ and $R_{c,I}^{u}$ is 1 because all D2D users and the cellular users share the same spectrum non-orthogonally.
- The sum capacity when the new D2D paper is in the overlay mode is given by
  (24) $$\begin{array}{cc} R_{o}^{u} & =\frac{N}{N+1}\cdot \underset{\mathrm{existing}\;D2D\;\mathrm{pairs}}{\underset{︸}{R_{d}}}\\ & +\frac{1}{N+1}\cdot \underset{\mathrm{new}\;D2D\;\mathrm{pair}}{\underset{︸}{\log(1+{\mathrm{SINR}}_{UN,ov})}}+\frac{N}{N+1}\cdot \underset{\mathrm{BS}}{\underset{︸}{R_{c,I}}}, \end{array}$$
  where $R_{d}=\log\left(\det\left(I_{M}+\beta_{M}H_{d}^{\prime }Q_{M}H_{d}^{\prime H}\right)\right)$ denotes the capacity of existing D2D pairs and $H_{d}^{\prime }={\left[h_{1},\ldots ,h_{M}\right]}^{T}\left[w_{d,1},\ldots ,w_{d,M}\right]$. $Q_{M}=V_{M}SV_{M}^{H}$ denotes the existing D2D pairs’ signal covariance, each column of $V_{M}$ is an eigenvector which can be obtained by SVD of the channel matrix $H_{d}^{\prime }$: $U_{M}D_{M}V_{M}^{H}=H_{d}^{\prime }$. $R_{c,I}=\log(\det(I_{N_{B}}+\mu H_{B}Q_{c}H_{B}^{H}))$ denotes the capacity of BS and μ is the SINR of BS. The interference in μ comes from existing D2D pairs. The coefficient of each term in (24) can be deduced with the aid of Figure 4.
- The sum capacity when the new D2D paper is in the cellular mode is given by
  (25) $$\begin{array}{c} R_{c}^{u}=\frac{N}{N+1}\cdot \underset{\mathrm{existing}\;D2D\;\mathrm{pairs}}{\underset{︸}{R_{d}}}\\ \;+\frac{1}{N+1}\cdot (\frac{1}{2})\cdot \underset{\mathrm{new}\;D2D\;\mathrm{users}}{\underset{︸}{\log(1+{\mathrm{SINR}}_{UN,cel})}}+\frac{N}{N+1}\cdot \underset{\mathrm{BS}}{\underset{︸}{R_{c,I}}}, \end{array}$$
  where $R_{d}$ and $R_{c,I}$ is the same as the definition in overlay mode and coefficient of each term in (25) can be seen with the help from Figure 4.

Next, when the existing D2D pairs are in the overlay mode, the sum capacity of the whole cell with the new D2D pair in the underlay, overlay, and cellular modes are respectively given as follows.

- The sum capacity when the new D2D paper is in the underlay mode is given by
  (26) $$\begin{array}{cc} R_{u}^{o} & =\left(1-\alpha \right)\cdot R_{d}\\ & +\alpha \cdot \log(1+{\mathrm{SINR}}_{OV,un})+\alpha \cdot \underset{\mathrm{BS}}{\underset{︸}{R_{c,I}^{o}}}, \end{array}$$
  where $R_{c,I}^{o}=\log(\det(I_{N_{B}}+\eta H_{B}Q_{c}H_{B}^{H}))$ denotes the capacity of BS and η is the SINR of BS. The interference in η comes from the new D2D pair.
- The sum capacity when the new D2D paper is in the overlay mode is given by
  (27) $$\begin{array}{cc} R_{o}^{o} & =(\frac{M}{M+1}\cdot \left(1-\alpha \right))\cdot \underset{\mathrm{existing}\;D2D\;\mathrm{pairs}}{\underset{︸}{R_{d}}}\\ & +(\frac{1}{M+1}\cdot \left(1-\alpha \right))\cdot \underset{\mathrm{new}\;D2D\;\mathrm{pair}}{\underset{︸}{\log(1+{\mathrm{SINR}}_{OV,ov})}}\\ & +\alpha \cdot \underset{BS}{\underset{︸}{R_{c}}}, \end{array}$$
  where $R_{c}=\log(\det(I_{N_{B}}+\epsilon H_{B}Q_{c}H_{B}^{H}))$ denotes the capacity of BS and ϵ is the SNR of BS.
- a new D2D pair in cellular mode
  (28) $$\begin{array}{c} R_{c}^{o}=(\frac{M}{M+1}\cdot \left(1-\alpha \right))\cdot \underset{\mathrm{existing}\;D2D\;\mathrm{pairs}}{\underset{︸}{R_{d}}}\\ \;+(\frac{1}{2(M+1)}\cdot \left(1-\alpha \right))\cdot \underset{\mathrm{new}\;D2D\;\mathrm{pair}}{\underset{︸}{\log(1+{\mathrm{SINR}}_{UN,cel})}}\\ \;+\alpha \cdot \underset{\mathrm{BS}}{\underset{︸}{R_{c}}}, \end{array}$$
  where $R_{d}$ and $R_{c}$ have previously been defined in (24). The best mode for the new pair of D2D users is selected by the highest sum rate:
  (29) $${\mathrm{Mode}}_{UN}=\underset{mode}{\mathrm{argmax}}R_{mode}^{u},$$
  (30) $${\mathrm{Mode}}_{OV}=\underset{mode}{\mathrm{argmax}}R_{mode}^{o}.$$

## 5. Simulations

We conduct numerical simulations in this section to demonstrate the effectiveness of the mode selection rules discussed in the previous sections. In the simulations, the number of cellular users is N=4 and the number of existing D2D pairs M=3. We randomly assign the distances between the transmitter and receiver in each D2D pair and between each D2D receiver and cellular user in [0,10] meters, with uniform distribution. The number of cellular users’ total transmit antennas is $M_{t}=8$. The path loss exponent is v=4. As a more reasonable setting, we set a longer distance from the BS to each of the cellular users or D2D users, which follows the uniform distribution in [0,100] meters. We compare the performance in SINR and in sum capacity of the proposed selection rules for the new pair of D2D users with a different set of cooperative precoding schemes, as discussed in Section 3.

### 5.1. Current Active D2D Pairs in the Underlay

**Figure 6:**
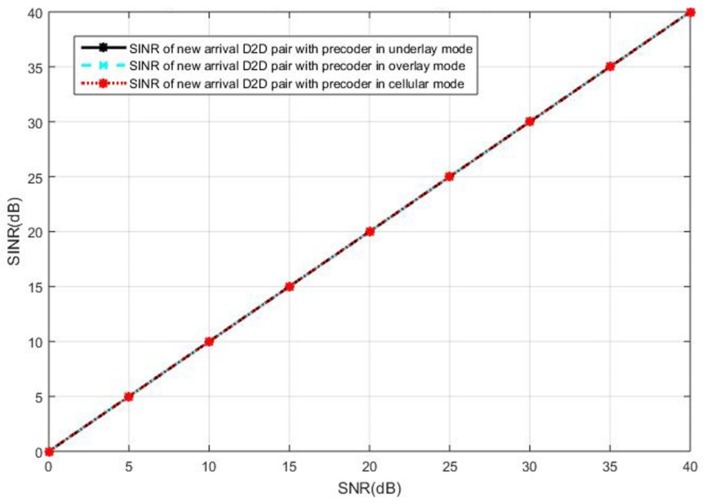
The intended receiver’s signal-to-interference-plus-noise-ratio (SINR) performance in the new pair of D2D users with the block diagonalization (BD) precoding for the celluar users and zero-forcing (ZF) precoding for the D2D users, in the case of current active D2D pairs operating in underlay.

We first present the simulations results when the current active D2D users operate in the underlay mode. The SINR observed at the intended receiver in the new pair of D2D users is shown in Figure 6, when the cellular users cooperatively employ BD precoding and the D2D users cooperatively employ the ZF precoding. It can be seen in Figure 6 that the interference toward the new D2D user is completely eliminated, as the SINR is equal to SNR in all curves, by the BD precoding in the cellular users and the ZF precoding in the D2D users. In this case, we see the same performance for the three selection modes. However, the ZF precoding suffers from the limitation of antenna constraints, as discussed in Section 3.

**Figure 7:**
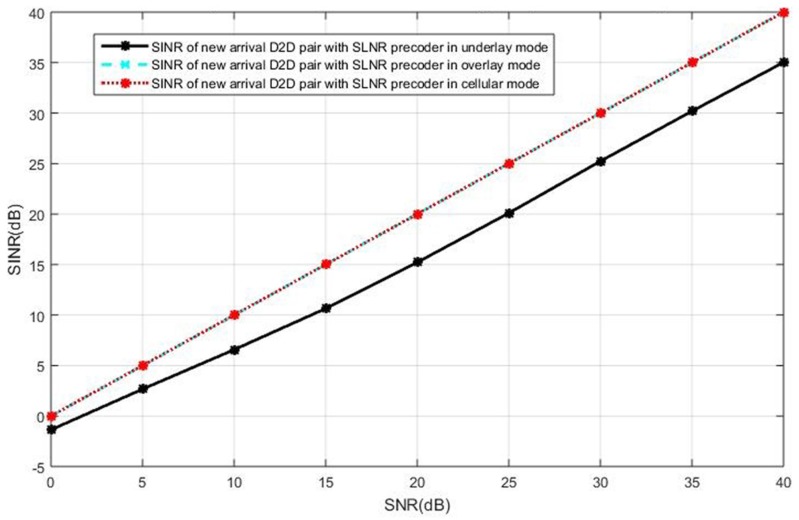
The intended receiver’s SINR performance in the new pair of D2D users with the SLNR precoding for both the cellular and D2D users, in the case of current active D2D pairs operating in underlay.

The SINR observed at the new D2D receiver is shown in Figure 7 when both the cellular users and the D2D users employ the SLNR precoding, which poses no constraints on the number of antennas. In Figure 7, we see the intended user of the new D2D pair in the underlay mode receives less SINR than that in the cellular or overlay mode. This is because the SLNR precoding cannot completely remove the interference in the case of the new D2D receiver operating in the underlay mode, in which the interference impacts on the SINR due to non-orthogonal resources reuse while the new D2D user suffers no interference in the overlay and the cellular mode. In this case, the overlay mode and the cellular mode are the better options compared with the underlay mode.

**Figure 8:**
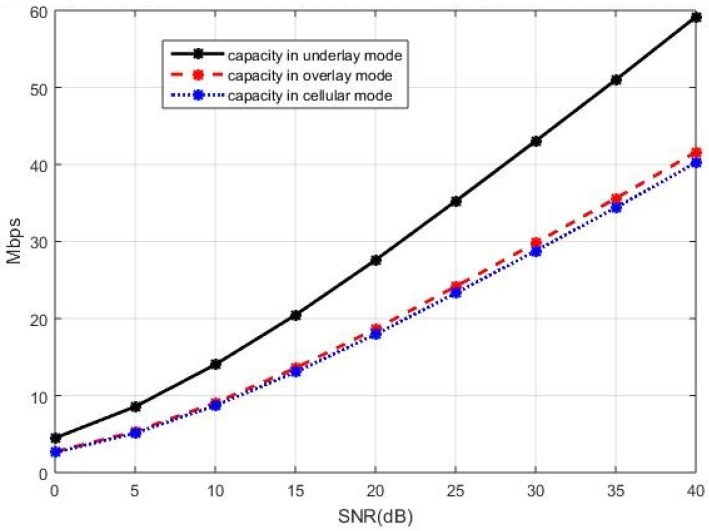
The sum capacity performance of the entire network with the ZF precoding and the BD precoding for the D2D and cellular users respectively, in the case of the current active D2D users operating in the underlay mode.

**Figure 9:**
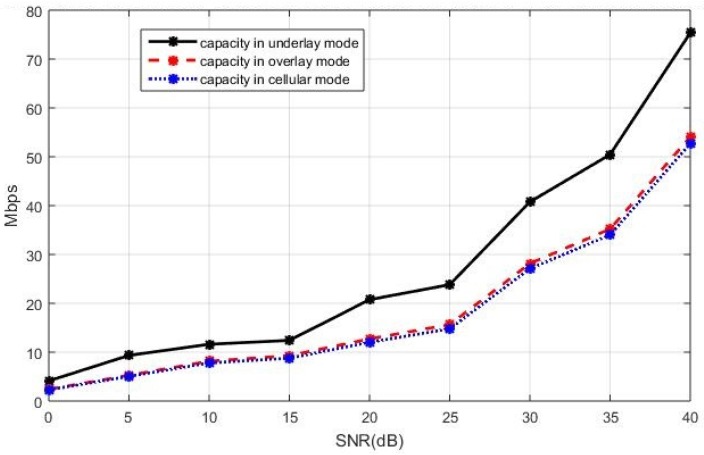
The sum capacity performance of the entire network with the SLNR precoding for both the celluar users and the D2D users, in the case of the current active D2D users operating in the underlay mode.

The sum capacity of the network using the capacity-based selection rule is presented in Figure 8 when the cellular users cooperatively employ the BD precoding and the D2D users cooperatively employ the ZF precoding. In Figure 8, we see that the underlay mode has a better sum capacity compared with the overlay and cellular modes. A similar performance trend can also be observed in Figure 9, where the sum capacity of the network is plotted with the SLNR precoding implemented in both the cellular users and the D2D users. This is because, in the underlay mode, the existing active D2D users and the new D2D user are allowed to utilize all available spectrum when coexisting with the cellular users while, in the overlay and in the cellular mode, each D2D user only utilizes a portion of the available spectrum in an orthogonal fashion. With properly controlled interference by the BD precoding for the cellular users and by the ZF precoding for the D2D users, the more available spectrum benefits to the sum capacity when the new D2D user operates in the underlay mode. In this case, the underlay mode performs better compared with the other two modes.

### 5.2. Current Active D2D Pairs in the Overlay

**Figure 10:**
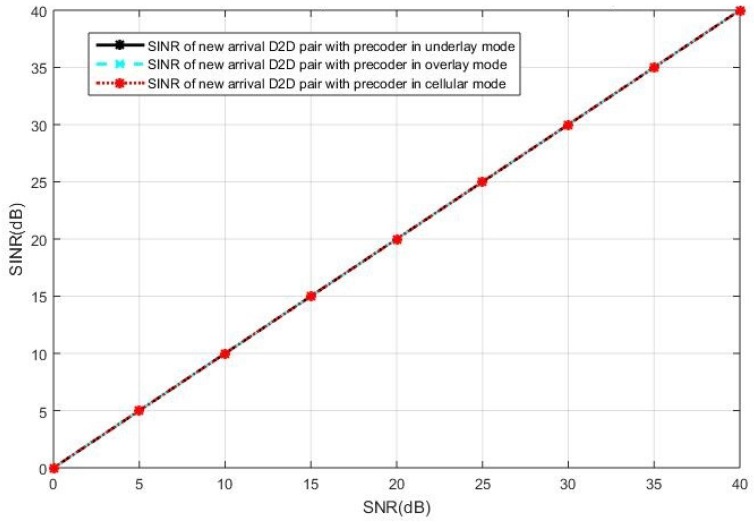
The intended receiver’s SINR performance in the new pair of D2D users with the ZF precoding and the BD precoding for the D2D and the cellular users, respectively, in the case of the current active D2D pairs operating in the overlay mode.

**Figure 11:**
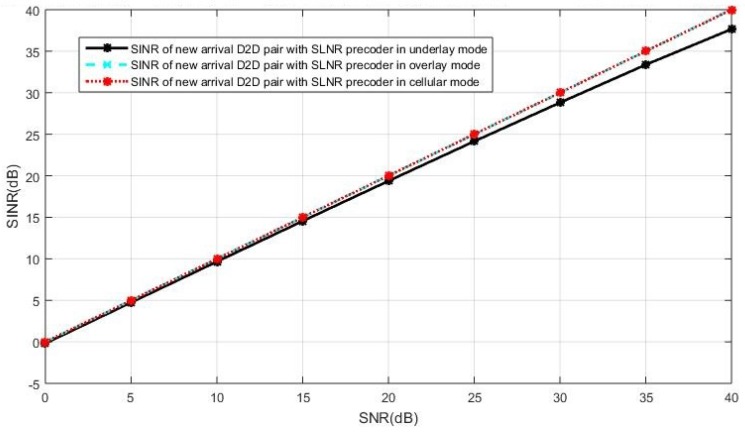
The intended receiver’s SINR performance in the new pair of D2D users with the SLNR precoding for both the D2D and the cellular users, in the case of the current active D2D pairs operating in the overlay mode.

We in this subsection present the simulations results when the existing D2D users operate in the overlay mode. Figure 10 shows the SINR observed at the intended receiver of the new D2D pair with the SINR-based selection rule, where the cellular users cooperatively employ the BD precoding. Note that the existing D2D users do not have to initiate any precoding as they operate in the overlay mode. It can be seen in Figure 10 that the interference toward the new D2D user from the cellular users is mitigated by the BD precoding in the cellular users. In this case, we see the same performance for the three selection modes. On the other hand, we find in Figure 11 that the overlay and the cellular modes both perform slightly better than the underlay mode. The difference between the underlay mode and the overlay/cellular modes is not as obvious as that in Figure 7. This is because the interference in the case of Figure 10 is from the cellular users only.

**Figure 12:**
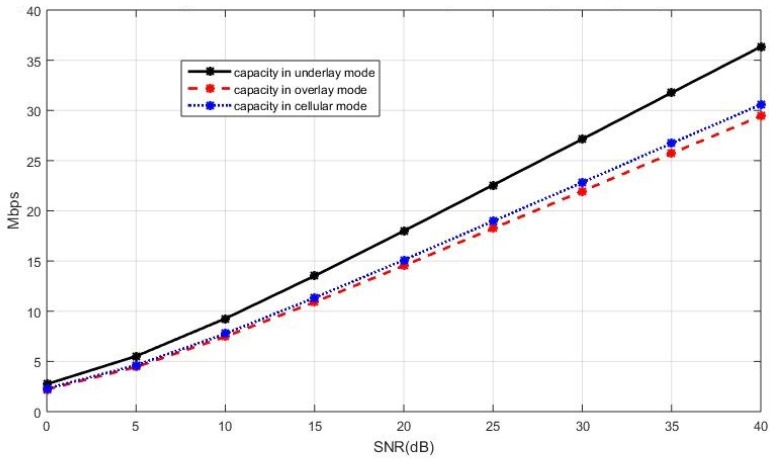
The sum capacity of the network with the BD precoding for the cellular users, in the case of the existing D2D users operating in the overlay mode.

**Figure 13:**
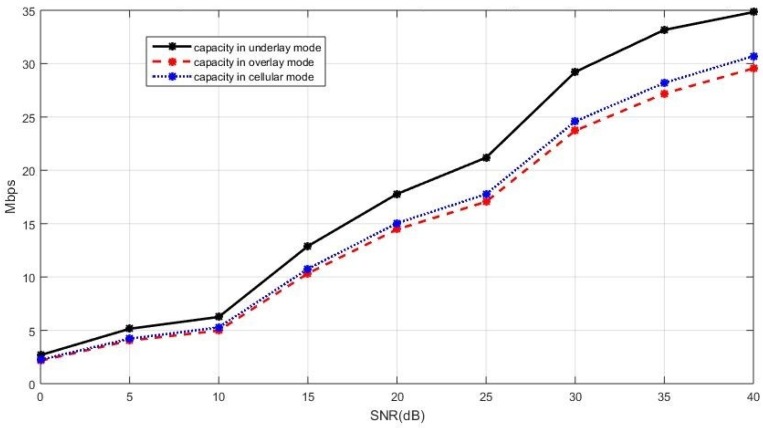
The sum capacity of the network with the SLNR precoding for the cellular users, in the case of the existing D2D users operating in the overlay mode.

We set α=0.5, the proportion of the spectrum used by the cellular users, in simulating the curves here. The sum capacity of the cellular network coexisting with multiple D2D users with the BD precoding and the SLNR precoding is presented in Figure 12 and Figure 13, respectively, where the mode selection is based upon the sum capacity. The trend of the simulation results here is similar to those appeared in Figure 8 and Figure 9, where current active D2D pairs are considered in the underlay mode. The sum capacity of the network with the new D2D pair adopting the underlay mode is much higher than that adopting the overlay and the cellular modes.

**Figure 14:**
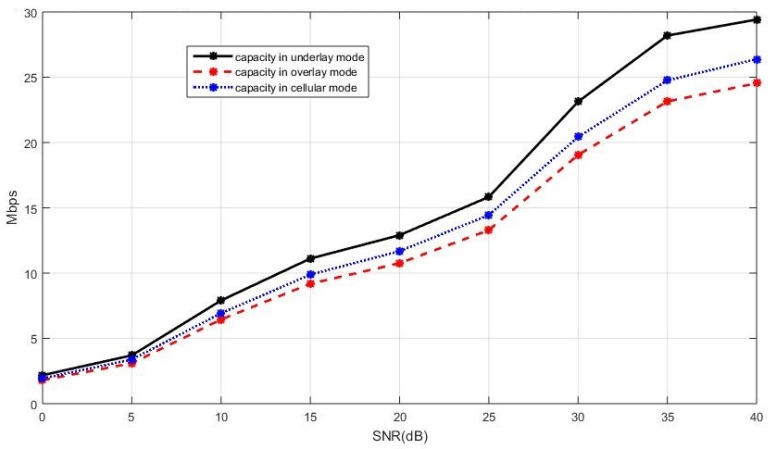
The sum capacity of the whole network with the SLNR precoding and α=0.2.

**Figure 15:**
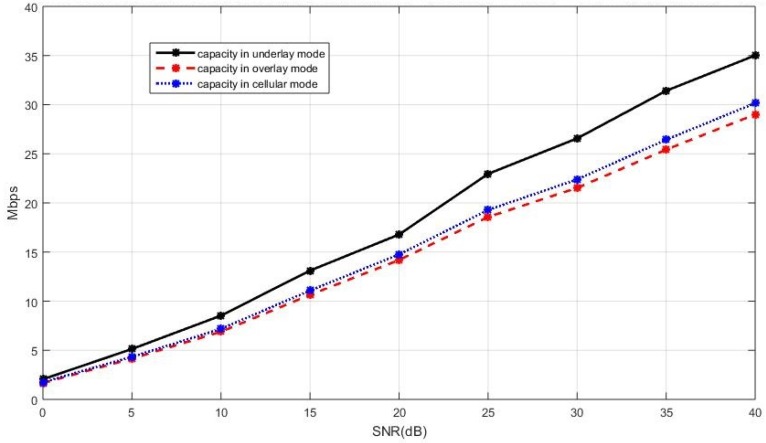
The sum capacity of the whole network with the SLNR precoding and α=0.5.

**Figure 16:**
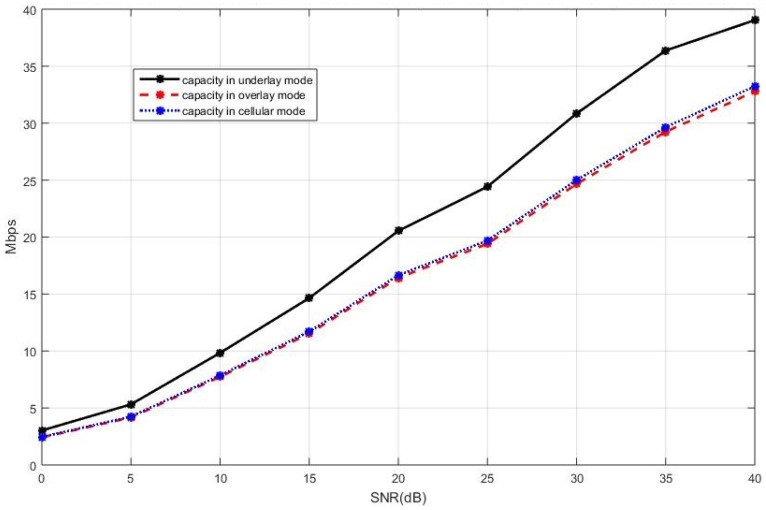
The sum capacity of the whole network with the SLNR precoding and α=0.8.

Finally, we discuss the sum capacity of the network with different values of α when the existing D2D pairs are in the overlay mode and the cellular users implement the SLNR precoding. The results of the sum capacity are shown in Figure 14, Figure 15 and Figure 16, respectively, with α=0.2, 0.5, 0.8. It can be observed that the maximum of the achievable sum capacity increases as α increases, which implies that the cellular users receive more resources. Moreover, the number of cellular users is more than the sum of a new D2D pair plus multiple existing D2D users.

## 6. Conclusions

We have studied the mode selection strategies for a new D2D pair becoming active in a network with a number of active D2D pairs coexisting with cellular users in a heterogeneous network. Specifically, the selection rules based on the SINR and the sum capacity combined with different precoding schemes have been investigated under a variety of scenarios. From the simulations, we have justified that, with the CSI available at the transmitters and the number of antennas satisfying the feasibility constraint of ZF precoding, the interference imposed upon the new D2D user can be removed using the BD and ZF precoding. As an alternative to the BD/ZF precoding, it has been shown analytically in [35] that the SLNR precoding needs not the constraint on the number of antennas while enjoying comparable performance to the BD/ZF precoding method. We note that the two selection rules, SINR-based and the capacity-based, considered in this paper impact on the system differently, with interesting tradeoffs presenting from different perspectives, where the SINR-based selection is primarily beneficial to the new individual D2D receiver while the capacity-based selection aims at lifting the performance of the entire network. Simulation results have provided several insights into the best selection among the modes depending on a variety of use cases in the network.
